# Bone Union and Safety of the Long Chimaera Nail in Extracapsular Femoral Fractures: A Retrospective Multicenter Study

**DOI:** 10.7759/cureus.110429

**Published:** 2026-06-08

**Authors:** Armando Del Prete, Giacomo Errico, Giuseppe Iodice, Roberto Civinini

**Affiliations:** 1 Department of General Orthopedic, University of Florence, Azienda Ospedaliero-Universitaria Careggi, Florence, ITA; 2 Department of Emergency and Admissions, Orthopedics and Traumatology Unit, AORN "Sant'Anna e San Sebastiano", Caserta, ITA

**Keywords:** bone union, chimaeratm cephalomedullary nail, complications, extracapsular proximal femoral fractures, hip fracture fixation, retrospective study

## Abstract

Background: Proximal femoral fractures (PFFs) are a major source of morbidity and mortality in the elderly. Although cephalomedullary nailing (CMN) systems are widely used for the treatment of extracapsular/trochanteric PFFs, the optimal implant choice remains debated - in particular, in the case of stable trochanteric fracture patterns. Chimaera^TM^ (Orthofix, Italy) is a novel CMN system designed to enhance fracture compression and rotational stability through a telescoping lag screw and optional anti-rotational element. This retrospective, multicenter, post-market study evaluated the clinical benefit and safety of the long Chimaera^TM^ nail in adults with extracapsular PFFs in a real-world setting.

Methods: The study cohort included 44 patients (mean age: 74.0 ± 14.5 years; 68.2% female), whose clinical data were assessed to evaluate bone union within 12 months, as well as adverse events and complications.

Results: Among patients included for union assessment evaluation (n=42), eventual union was clinically documented in all cases (100%; 95% CI: 91.6-100%); in line with the retrospective design and a variable imaging availability, this estimate refers to evaluable patients only, with X-ray documented union in 29 patients. Overall, 38.1% of evaluable patients had clinically documented union at first follow-up (1.3 months) and a median time to documented union of 7.4 months. Four adverse events (9.1%) and two reoperations (4.5%) were observed, none related to the device. No implant-related fractures or screw cutouts were reported, and no deaths were recorded. Mean operative time was 91.3 ± 45.7 minutes, and mean tip-apex distance (TAD) decreased from 27.2 mm postoperatively to 24.7 mm at late follow-up.

Conclusions: Despite inherent limitations due to the retrospective design and the lack of a control group, results confirm the favorable benefit-risk profile of the Chimaera^TM^ long nail in a real-world setting, showing high consolidation rates and a low incidence of complications.

## Introduction

Proximal femoral fractures (PFFs) are a leading cause of severe morbidity and mortality in the elderly, triggering immediate mechanical impairment and implying systemic complications [1,2]. More than 1.3 million cases per year are estimated globally [3], and, as the population ages, the annual number of hip fractures is expected to nearly double by 2050 compared with the 983,056 cases estimated in 2018 [1,2]. Around 90% of PFFs happen in subjects over 50 years and usually after low-energy injuries, while only 2% occur in young adults, who mostly report high-energy traumas [4,5]. With age, indeed, osteoporotic processes elicit trabecular structure degeneration in the proximal femur, which leads to loss of integrity and a consequent higher likelihood of fracture [6].

Bone healing is one of the main clinical challenges in the treatment of PFFs. In case of extracapsular fractures, this objective can be difficult to achieve due to osteoporotic bone quality, posteromedial comminution, and the need for stable fixation allowing controlled impaction. Cephalomedullary nailing (CMN) - i.e., the intramedullary fixation inserted through the greater trochanter, aligned with the femoral neck axis [7] - is a mainstay among the operative treatment options currently available because of its load-sharing construct and favorable biomechanics [8,9]. Modern CMN systems incorporate a sliding lag screw that allows controlled axial impaction under weight-bearing, thereby promoting physiological fracture healing and lowering shear forces at the fracture site [10,11]. Clinical evidence exists showing that treatment of extracapsular PFFs with CMN can allow achieving average bone union rates of ~98%, with low reoperation rates in older adults [11-13]. Furthermore, advantages include shorter operation times, reduced intraoperative blood loss, smaller incisions, shorter hospital stays, and improved functional outcomes. On the other hand, events of cut-out, lateral protrusion, Z-effect, and secondary fractures still represent possible complications and remain a target for optimization of CMN systems [14,15].

While high-level evidence is already available about clinical advantages and limitations associated with CMNs in the treatment of extracapsular PFFs, the preferential use of short versus long nails is still debated. The former may be more cost-effective, require less operating room time, and favor reduced blood loss, but their shorter diaphyseal fixation has been associated with thigh pain and peri-implant fractures at the distal tip. On the other hand, long nails can provide more extensive diaphyseal fixation and may be associated with a reduced risk of distal femoral shaft/peri-implant fractures [16].

The Chimaera^TM^ Hip Fracture System (Orthofix, Bussolengo, Italy) - hereafter Chimaera^TM^ - is a novel intramedullary device indicated for the treatment of stable and unstable PFFs and manufactured either as a short or long nail. Chimaera^TM^ features a telescoping lag screw housed within a fixed barrel, with an optional supplementary telescoping/anti-rotational screw. This design helps mitigate rotational instability in the femoral head while ensuring smooth sliding of the lag screws during weight-bearing, eventually favoring controlled compression. Real-world evidence concerning the use of this CMN system, though, is still limited.

To address this gap in clinical evidence, we report results from a retrospective, real-world investigation conducted to further explore the benefit-risk profile of the Chimaera^TM^ long nail in routine clinical practice.

## Materials and methods

Study design 

This was a retrospective, observational, single-arm, multicenter study evaluating clinical benefit and safety of Chimaera^TM^ (Orthofix, Bussolengo, Italy) in real-world practice. The study was designed within the framework of post-market surveillance activities under the provisions of the MDR 745/2017. The study started on November 27, 2023, and was completed on December 18, 2024, involving two centers in Italy - Azienda Ospedaliero Universitaria Careggi (Florence) and Azienda Ospedaliera di Caserta - A.O.R.N. S. Anna e S. Sebastiano (Naples). It was approved by the institutional ethics committees of each site and was conducted in accordance with guidelines for Good Clinical Practice concerning clinical investigations on human subjects, the Declaration of Helsinki, and ISO 14155:2020. All participants agreed to participate in the study; written informed consent was collected retrospectively for data use.

Patients

All consecutive eligible patients treated during the study period at the participating centers were screened for study inclusion. Eligible patients were adults with regular indication for surgical intervention with Chimaera^TM^ long nail according to the manufacturer's instructions (i.e., treatment of stable or unstable pertrochanteric, intertrochanteric, and subtrochanteric femoral fractures, either isolated or associated with distal shaft extension of different etiology), sufficient clinical data to assess the safety and efficacy endpoints of the study, and who signed the informed consent. Exclusion criteria were contraindication to the use of the device according to the manufacturer's instructions; bilateral proximal femur fractures; application of a concomitant not permitted device, which could not be safely removed; concurrent medical or non-medical conditions that, in the opinion of the investigator, may have prevented participation or otherwise rendered the patient ineligible for the study; and participation to other clinical studies (or participated to clinical studies within three months prior signing the informed consent).

Device and surgical procedure

Chimaera^TM^ consists of an internal cephalomedullary fixation system intended to treat individuals suffering from stable and unstable pertrochanteric, intertrochanteric, and subtrochanteric fractures. It is manufactured either as short or long nailing, with lengths ranging from 280 mm to 460 mm, in 20 mm increments. The fixation system is made of sterile titanium alloy with anodized type II surface treatment and is cannulated for guidewire-controlled insertion. The proximal diameter of the nail is 15.5 mm; the distal diameter is 10 mm and 11 mm. There are two proximal nail caput-collum-diaphyseal angles available: 125° or 130°. The implantable components include the nail, end cap, lag screw, threaded locking screw, and optional supplementary lag screw. Materials such as stainless steel, aluminum alloy, carbon fiber, composite, and plastic instrumentation refer to non-implantable components. The lag screw locks itself into the nail. The dynamic distal locking hole can be used to allow fracture compression up to 6 mm in the diaphysis direction.

Surgeons were experienced with the system and followed routine site practice during intervention. All patients received the long version of the Chimaera^TM^ nail, as per standard practice at the participating centers. Short nails were not used.

Clinical assessment and follow-up schedule

Patients were retrospectively screened from medical records at each site. Demographic, clinical, and safety data were collected from the time of surgery until the last follow-up visit, including laterality and mechanism of injury. Assessments reflecting routine care were conducted on the day of surgery, at hospital discharge, at the first follow-up visit for bone consolidation assessment, and at two additional follow-up visits. The study protocol defined windows of one month for the first follow-up visit and 3-9 months and 9-15 months for the second and third follow-up visits after surgery, respectively; however, due to the retrospective design, some visits could have occurred outside these windows. Therefore, while protocol labels for follow-up visits refer to the planned assessment windows, the analyses were conducted considering the actual timing of available follow-up assessments.

Radiographic imaging and fracture classification

Radiographic imaging included anteroposterior (AP) and lateral (LAT) intraoperative fluoroscopy or early postoperative radiographs documenting the final nail placement, and AP+LAT radiographs at follow-up, when available.

Fracture morphology was classified according to the AO Foundation/Orthopaedic Trauma Association (AO/OTA) system for proximal femur fractures (31-A) [17], as recorded in the site medical records. The AO/OTA classification is a widely accepted universal alphanumeric coding system presented with schematic drawings and short explanations. The AO/OTA system for proximal femoral fractures (region 31) categorizes injuries based on anatomical location and stability, primarily into type A (trochanteric), type B (femoral neck), and type C (femoral head) fractures. Laterality (left/right) was captured from the surgical records, and the mechanism of injury (low vs high energy) was recorded as part of baseline characteristics.

Tip-to-apex distance (TAD; i.e. the sum of the distances from the tip of the cephalic screw to the apex of the femoral head, measured on both AP and LAT radiographs, after correction for radiographic magnification) was derived combining the recorded components of tip distance in AP view (X_AP), tip distance in lateral view (X_LAT), diameter in AP view (D_AP), and diameter in lateral view (D_LAT). Because imaging availability could have varied across visits in this retrospective setting, radiographic parameters (including TAD) were summarized only for cases with complete AP+LAT data.

Bone union assessment

Bone union was determined from the medical records and classified as eventual clinically documented union because assessment relied either on radiographic evidence or on clinical evaluation of the treating team, and at follow-up windows that possibly differed from those initially planned. When radiographs were available at follow-up, union was assessed independently by two experienced physicians according to standard clinical practice, considering serial radiographic evidence of fracture healing and the patient's clinical evolution; no formal quantitative radiographic scoring system or predefined threshold was defined in the protocol, and assessors were not formally blinded. Radiographic union was confirmed only when both observers agreed. Since no disagreements occurred, no adjudication procedure was required; formal inter-observer reliability testing was not prospectively planned. In the absence of imaging, union assessment was based on the treating team's evaluation reported in the Case Report Form (CRF), which included a telephone follow-up assessment. Considering the retrospective nature of the study, some follow-up visits were conducted by telephone and/or occurred outside protocol windows. Consequently, the recorded time to bone consolidation reflects the earliest visit at which union was clinically documented by the treating team and/or radiographically confirmed by both readers when images were available.

Objectives and endpoints

The primary objective was to evaluate the clinical benefit of the long variant of Chimaera^TM^ used in adult patients. The primary endpoint was the proportion of patients achieving bone union within 12 months of implantation. Patients were included in the primary objective assessment only if they did not present contralateral fractures or refractures at the treated site. Patients reoperated for secondary dynamization, with reoperation after an initial union assessment, and with upper-limb fractures unrelated to the device, were included in the analysis.

The secondary objective was evaluation of safety, defined as the proportion of patients requiring reoperation due to adverse device effects (ADE/SADE) or medical device deficiency (MDD) from implantation, including events that occurred or could reasonably have led to reoperation (e.g., cut-out not operated for external reasons). Safety events were classified according to the study protocol. Adverse events (AEs) included any untoward medical occurrence irrespective of device causality, whereas serious AEs (SAEs) were events resulting in death, life-threatening condition, hospitalization, or prolongation of hospitalization, disability/incapacity, or medical/surgical intervention. ADEs and SADEs were defined as AEs or SAEs for which a causal relationship with the investigational device or its use could not be excluded. MDDs were defined as inadequacies related to device identity, quality, durability, reliability, safety, or performance, including malfunction, use error, or inadequacy of information supplied by the manufacturer. Event seriousness and causality were attributed by the investigator based on the evaluation of medical records.

Analysis of TAD, as well as of benefit and safety outcomes in patients classified according to the AO/OTA fracture subtype, was included as exploratory endpoints.

Statistical analysis

The target sample size (n = 44) was selected from a sensitivity analysis based on a reference union rate of ~98%, aiming for a 5% imprecision in the estimate. All enrolled patients comprised the full analysis set (FAS) for safety.

Continuous variables were described with measures of central tendency and dispersion, including mean, median, standard deviation (SD), and interquartile range (IQR: Q1-Q3), minimum, and maximum. Discrete variables were described using absolute and relative frequencies. In the descriptive analysis of qualitative variables, both total percentage and valid percentage were considered, representing, respectively, the percentage over the sum of valid responses plus missing values and the percentage over the total of valid responses. Clopper-Pearson confidence intervals (CI) are shown at 95.0%. Analyses were conducted on the FAS for safety, while efficacy was estimated on patients meeting the inclusion criteria for assessment of the primary objective. Missing data were not imputed. Data were analyzed using Statistical Product and Service Solutions (SPSS, version 29.0; IBM SPSS Statistics for Windows, Armonk, NY).

## Results

Study population

**Figure 1:**
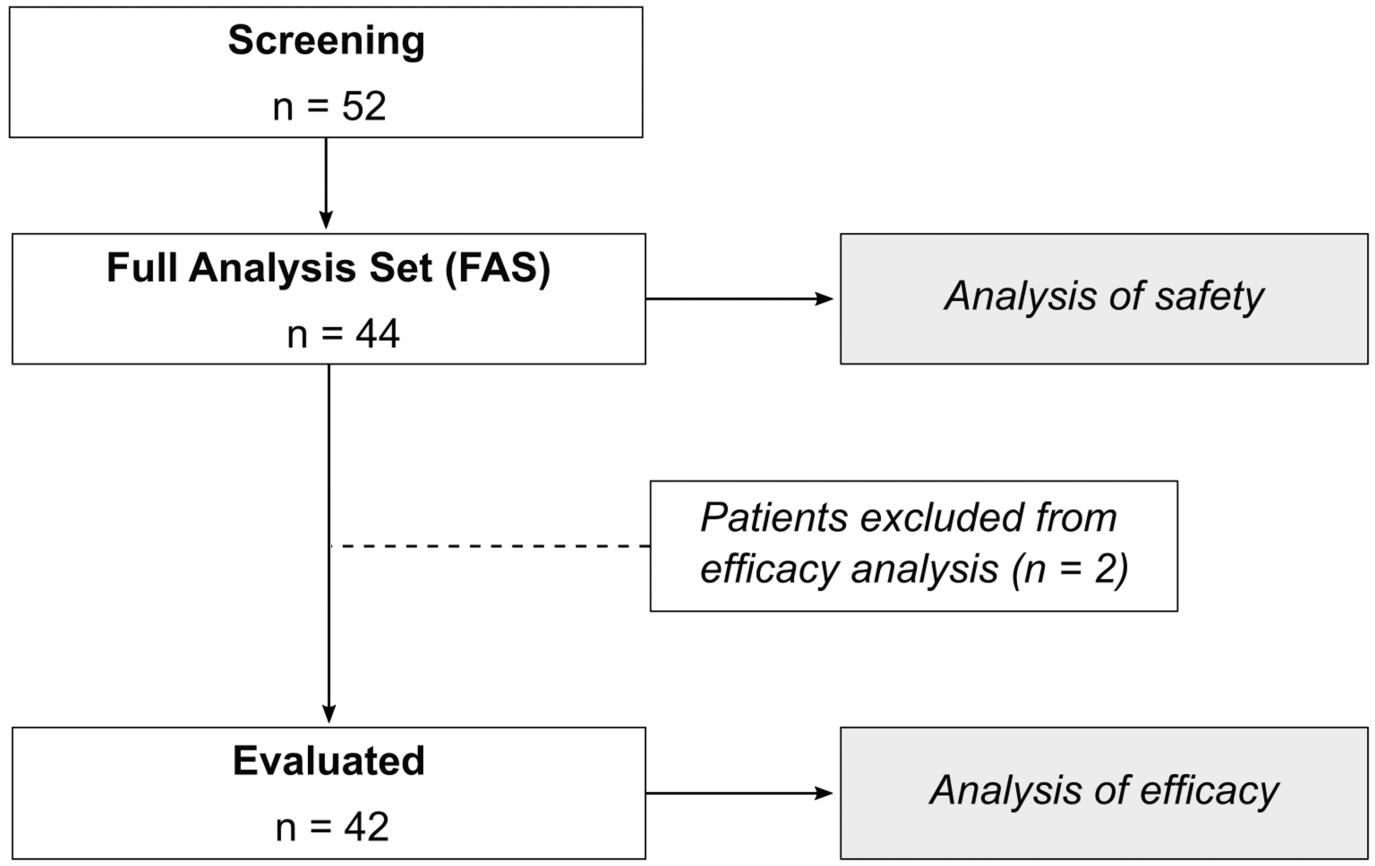
Schematic representation of patient disposition for safety and efficacy analyses.

In total, 52 patients were screened. Eight subjects were classified as screen failures and were not enrolled: two patients died before the collection of follow-up data, four patients did not present sufficient clinical data to allow evaluation of study endpoints in accordance with the study protocol, and two additional patients decided to withdraw consent to participate after initial signature. Overall, 44 were enrolled and constituted the FAS, with no reported discontinuation (Figure 1).

An overview of demographic and clinical characteristics at baseline is provided in Table 1.

**Table 1:** Demographic characteristics of patients included in the full analysis set (FAS, N = 44) after initial screening according to the inclusion and exclusion criteria. Statistical parameters include absolute and relative percentages (N, %), mean and standard deviation ± SD, median and interquartile range (IQR, Q1-Q3), and minimum to maximum range.

Variable	Category	N (%)	Mean ± SD	Median (IQR)	Range
Age (years)	-	44	74.0 ± 14.5	78.0 (67.1-84.1)	32.8-95.1
Sex	M	14 (31.8)	-	-	-
	F	30 (68.2)	-	-	-
Ethnicity	Caucasian	44 (100)			
Weight (kg)	-	16	72.9 ± 11.2	74.5 (61.3-80.0)	55.0-95.0
BMI (kg/m^2^)	-	16	26.6 ± 3.9	26.0 (23.5-30.1)	20.7-33.2
Employed	Yes	5 (11.4)	-	-	-
	No	22 (50)	-	-	-
	Missing	17 (38.6)	-	-	-
Smoke	Yes	5 (11.4)	-	-	-
	No	13 (29.5)	-	-	-
	Missing	26 (59.1)	-	-	-
Comorbidity	Yes	29 (65.9)	-	-	-
	No	15 (34.1)	-	-	-
Comorbidity type	Osteoporosis	11 (25)	-	-	-
	Diabetes	5 (11.4)	-	-	-
	Tumor	3 (6.8)	-	-	-
	Other	20 (45.5)	-	-	-
Medication	Yes	15 (34.1)	-	-	-
	No	29 (65.9)	-	-	-

All patients were Caucasian and mostly female (68.2%). Mean age was 74.0 ± 14.5 years (range: 32.8-95.1), and mean BMI was 26.6 ± 3.9 kg/m^2^. Most patients (29/44: 65.9%) had comorbidities, mainly osteoporosis (11/29: 37.9%) and diabetes (5/29: 17.2%).

Diagnosis of fracture relied on X-ray in all cases; most (77.3%) were low-energy fractures. All 44 patients had traumatic, non-pathologic trochanteric fractures classified AO/OTA 31-A. Two patients also presented with shaft fractures (32-A1 and 32-B3), resulting in 46 total fractures treated during the same surgery. Implantation was on the left side on most occasions (61.4%). Nails with either 10- or 11-mm distal diameters were used. Lengths of implanted CMN ranged between 280 and 420 mm (347.3 ± 33.4 mm). All patients underwent distal locking, and 86.4% of them received a supplementary lag screw; 72.7% had static locking, 13.6% dynamic, and 13.6% secondary dynamization. Four patients (9.1%) required additional synthetic material for fracture treatment. Surgery lasted on average 91.3 ± 45.7 min. (35.0-330 min) (Table 2).

**Table 2:** Clinical data collected at clinical diagnosis, surgery visit, and discharge visit (N = 44). ^a^: "RX (AP + LAT) available" refers to the availability of standard postoperative or follow-up radiographs, including both anteroposterior and lateral views. ^b^: "Caput-Collum-Diaphyseal (CCD) angle" indicates the angle between the femoral neck axis and the femoral shaft axis. ^c^: "Fluoroscopy or RX (AP + LAT) available" indicates the availability of imaging documenting implant positioning obtained either intraoperatively (fluoroscopy) or postoperatively (standard AP and lateral radiographs). Statistical parameters include absolute and relative percentages (N, %), mean and standard deviation (SD), and median and interquartile range (IQR, Q1-Q3).

Variable	Category	N (%)	Mean ± SD	Median (IQR)
Clinical diagnosis				
Time from trauma to Informed Consent (IC) signature (days)	-	44	2.2 ± 1.5	2.0 (1.0-3.0)
Clinical fracture diagnosis	Non-pathological	44 (100)	-	-
Fracture classification	31 A 1.2	3 (6.8)	-	-
	31 A 1.3	16 (36.4)	-	-
	31 A 2.2	1 (2.3)	-	-
	31 A 2.3	10 (22.7)	-	-
	31 A 3.1	10 (22.7)	-	-
	31 A 3.3	4 (9.1)	-	-
Second fracture	Yes	2 (4.5)	-	-
	No	42 (95.5)	-	-
Fracture cause	Trauma	44 (100)	-	-
Fracture energy	High	7 (15.9)	-	-
	Low	34 (77.3)	-	-
	Spontaneous	1 (2.3)	-	-
	Missing	2 (4.5)	-	-
RX (AP + LAT) available^a^	Yes	44 (100)	-	-
Surgery visit				
Treated femur	Left	27 (61.4)	-	-
	Right	17 (38.6)	-	-
Distal nail diameter	10 mm	13 (29.5)	-	-
	11 mm	31 (70.5)	-	-
Caput-Collum-Diaphyseal (CCD) angle^b^	125°	30 (68.2)	-	-
	130°	14 (31.8)	-	-
Nail length (mm)	-	44	347.3 ± 33.4	340 (320.0-360.0)
Proximal lag screw	Fixed	25 (56.8)	-	-
	Sliding	19 (43.2)	-	-
Additional proximal screw	Yes	38 (86.4)	-	-
	No	6 (13.6)	-	-
Distal locking	Yes	44 (100)	-	-
Type of distal locking	Static	32 (72.7)	-	-
	Dynamic	6 (13.6)	-	-
	Secondary dynamization	6 (13.6)	-	-
Duration of surgery (min.)	-	44	91.3 ± 45.7	85.0 (65.0-100.0)
Transfusion	Yes	12 (27.3)	-	-
	No	32 (72.7)	-	-
Fluoroscopy or RX (AP + LAT) available^c^	Yes	43 (97.7)	-	-
	No	1 (2.3)	-	-
TAD (mm)	-	43	27.2 ± 8.5	26.3 (21.0-31.5)
Discharge visit				
Time from surgery to discharge (days)		44	7.2 ± 3.7	7.0 (5.0-8.8)
Medication after surgery	Yes	18 (40.9)	-	-
	No	26 (59.1)	-	-
Pain	Yes	15 (34.1)	-	-
	No	2 (4.5)	-	-
	Missing	27 (61.4)	-	-

Discharge and follow-up visits

Discharge visit was performed 7.2 ± 3.7 days (0-19 days) after surgery. The first follow-up visit was performed by 35 patients (51.4% outpatient visit, 48.6% contacted by phone) 1.3 ± 0.6 months (0.1-4 months) after surgery, in two cases outside the planned window. The second follow-up visit was performed by 10 patients (70% outpatient visit, 30% contacted by phone) 3.1 ± 0.9 months (1.9-4.6 months) after surgery, and five of them attended the visit outside the planned window. The third follow-up visit, planned within the 9-15-month protocol window, was attended by 29 patients (31% outpatient visit, 69% contacted by phone) 19.8 ± 18.5 months (3.3-73.4 months) after surgery, with 21 patients performing the visit outside the planned window (Table 3). Thus, the 9-15 months label refers to the planned protocol window, whereas the reported mean and range describe the actual timing of available follow-up assessments.

**Table 3:** Clinical data collected at first, second, and third follow-up visits. Statistical parameters include absolute and relative percentages (N, %), mean and standard deviation (SD), median and interquartile range (IQR, Q1-Q3), and minimum to maximum range. FU: Follow-up; TAD: Tip-apex distance

Variables		FU-1 (1 month), N =35	FU-2 (3-9 months), N = 10	FU-3 (9-15 months), N = 29
Time from surgery (months)	Mean ± SD	1.3 ± 0.6	3.1 ± 0.9	19.8 ± 18.5
	Median (IQR)	1.3 (1.1-1.4)	2.9 (2.5-4.0)	11.8 (7.6-26.2)
	Range	0.1-4.0	1.9-4.6	3.3-73.4
Visit in protocol window, N (%)	Yes	33 (94.3)	5 (50)	8 (27.6)
	No	2 (5.7)	5 (50)	21 (72.4)
Type of visit, N (%)	Outpatient	18 (51.4)	7 (70)	9 (31)
	Telephone	17 (48.6)	3 (30)	20 (69)
Pain, N (%)	Yes	3 (8.6)	4 (40)	0 (0)
	No	11 (31.4)	4 (40)	22 (75.9)
	Missing	21 (60)	2 (20)	7 (24.1)
Fluoroscopy or RX (AP + LAT) available, N (%)	Yes	35 (100)	-	18 (62.1)
	No	0 (0)	-	11 (37.9)
Bone consolidation (agreement by 2 observers), N (%)	Yes	16 (45.7)	-	28 (96.6)
	No	19 (54.3)	-	1 (3.4)
TAD (mm)	Mean ± SD	25.8 ± 8.7	-	24.7 ± 4.8
	Median (IQR)	25.2 (20.0-28.0)	-	24.9 (21.0-28.9)
	Range	12.6-48.3	-	14.7-31.5

Primary objective

The primary objective was evaluated on 42 patients satisfying the criteria for bone union assessment. Exclusion of two patients occurred because of insufficient follow-up data for union assessment, with no evidence of treatment failure in either case, in accordance with study protocol criteria. Consolidation was clinically confirmed in all 42 patients (100%, 95% CI: 91.6-100%) (Table 4), with X-ray documented union within 12-month follow-up confirmed in 29 (69%) of them. The endpoint was based on the earliest available documentation of union in the medical record, thus reflecting the eventual clinically documented union among evaluable patients rather than radiographically confirmed union within the predefined 12-month window. When documentation of union was available after 12 months, these patients were retained in the analysis, and timing was interpreted as delayed documentation due to missing imaging and/or out-of-window follow-up.

**Table 4:** Primary objective: eventual clinically documented bone union during available follow-up in evaluable patients (N = 42). ^#^: evaluation after excluding three outlier observations Statistical parameters include absolute and relative percentages (N, %), mean and standard deviation (SD), and median and interquartile range (IQR, Q1-Q3).

Variable	Category	N (%)	Mean ± SD	Median (IQR)
Bone consolidation (agreement by 2 observers)	Yes	42 (100)	-	-
	No	0 (0)	-	-
Time to bone consolidation after surgery (months)	-	42	13.6 ± 17.8	7.4 (1.3-20.8)
	-	39^#^	9.5 ± 10	7.1 (1.3-13.8)

**Figure 2:**
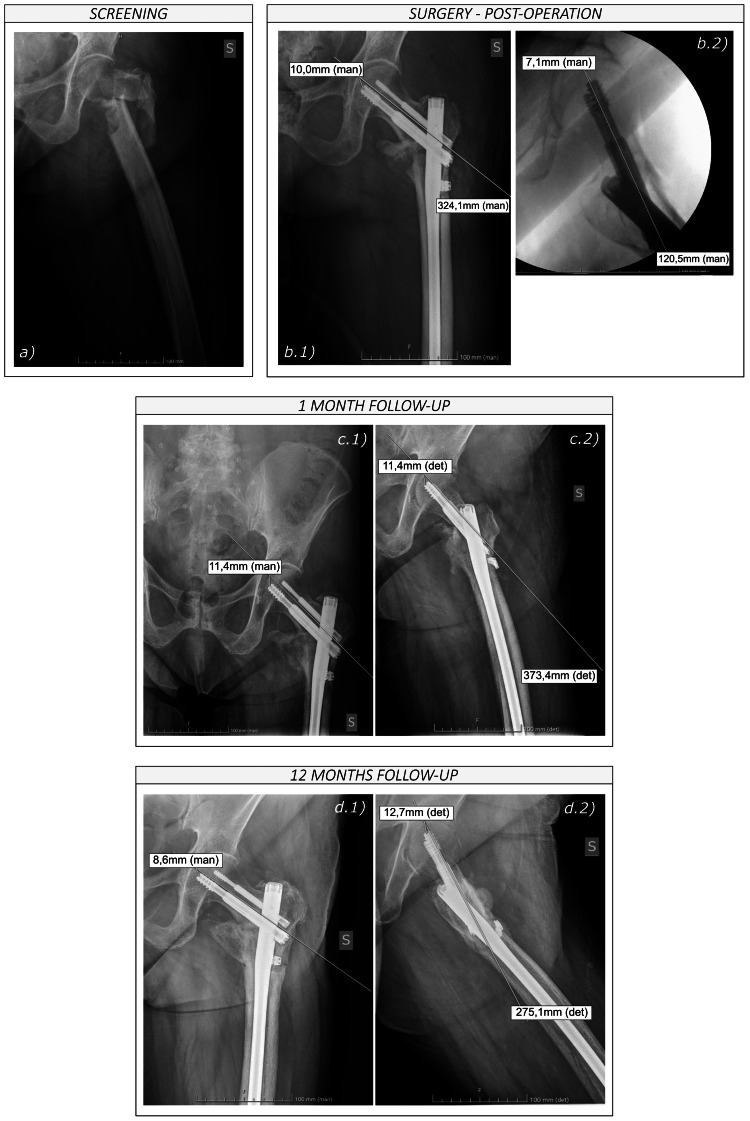
Radiographic course of a 72-year-old woman who sustained a low-energy left proximal femur fracture classified as AO/OTA 31-A2.3, comminuted and unstable. a) Preoperative anteroposterior radiograph obtained at screening. b.1-b.2) Immediate postoperative anteroposterior and lateral fluoroscopic images after fixation with a cephalomedullary nail with a 125° CCD angle and 380-mm nail length. The tip-to-apex distance (TAD) at surgery was 17.85 mm, calculated using the anteroposterior and lateral components shown on fluoroscopy (XAP = 10 mm; XLAT = 7 mm, automatically rounded by measurement software). c.1-c.2) One-month follow-up radiographs showing maintained implant position and early bone union; TAD was 23.1 mm. At this visit, the patient reported pain in the fractured hip. d.1-d.2) Twelve-month follow-up radiographs confirming fracture union and stable fixation; TAD remained 23.1 mm. The patient reported no hip pain at the second and final follow-up visits. No adverse events were recorded. AO/OTA: AO Foundation/Orthopaedic Trauma Association

Sixteen patients (38.1%) already achieved clinically documented bone union at the first follow-up visit, while the remaining 26 patients (61.9%) achieved bone union at the third follow-up visit (Figure 2). Median time to bone consolidation - i.e., the time from surgery to the first documented confirmation of union in the medical record - was 7.4 months in the 42 patients (IQR: 1.3-20.8 months); however, this measure should be regarded as the time to first available documentation of union in the medical record rather than the actual biological time to fracture healing. After exclusion of outlier measures (n = 3), median time to bone consolidation was 7.1 months (IQR: 1.3-13.8 months) (Table 4). Due to imaging variability and retrospective follow-up, the reported median time to consolidation reflects the first documented confirmation of clinically documented union rather than the biological healing time.

Safety outcomes

Adverse events (AEs) were evaluated in the FAS (44 patients). Four AEs occurred in four patients (9.1%) - including two serious events requiring additional surgery (4.5%) - and none related to the device or study procedure (Table 5).

**Table 5:** Secondary objective outcomes concerning adverse events and reoperations analyzed in the full analysis set (N = 44). ^#^: the events were not related to the device or caused by device deficiencies. Statistical parameters include absolute and relative percentages (N, %). FU: Follow-up

Variable	Category	N (%)
Adverse event (AE)	Yes	4 (9.1)
	No	40 (90.9)
Serious adverse event	Yes	2 (4.5)
	No	42 (95.5)
Adverse event requiring additional surgery	Yes	2 (4.5)^#^
	No	42 (95.5)
Type of AEs	Bone fracture	1 (25.0)
	Deep infection	1 (25.0)
	Anemia	1 (25.0)
	COVID infection	1 (25.0)
AE-related additional surgery	Yes	2 (4.5)
	No	42 (95.5)
Additional surgery follow-up	FU-1	1 (50.0)
	FU-2	1 (50.0)

Serious events occurred in two different patients. They consisted of one femoral shaft fracture that occurred 10 days after the first follow-up (it was treated with internal fixation and bone grafting) and one deep infection requiring nail removal and antibiotic therapy. The femoral shaft fracture, reported approximately 10 days after the first follow-up visit and before documented bone consolidation, occurred in the distal locking region of the nail; the fracture was treated with plate-and-screw fixation, cerclage, and bone graft support, while the Chimaera^TM^ nail was maintained in situ. According to the investigator's assessment, the event was attributed primarily to poor bone quality rather than to device malfunction, as no nail breakage, loosening, loss of fixation, or structural failure was observed. Therefore, the event was judged as not directly related to device performance.

The second SAE consisted of septic pseudoarthrosis that was not considered device-related.

One MDD was recorded, consisting of nail breakage. According to the investigator's assessment, though, the breakage occurred in the context of infection/septic non-union and was attributed to the patient's clinical condition rather than to an intrinsic device malfunction or structural defect. Therefore, no expected or unexpected adverse effects potentially or certainly related to Chimaera^TM^ were identified. No deaths were recorded, and no withdrawals occurred among patients in the FAS.

Exploratory outcomes

TAD had a mean value of 27.2 ± 8.5 mm at the end of surgery (N = 43) and 24.7 ± 4.8 mm at the third follow-up (N = 18), showing a modest reduction over time. No differential consolidation signals were observed for union or complications in patients classified according to AO/OTA subtype (31-A1/A2/A3).

## Discussion

In this real-world, retrospective study, we investigated the benefit-risk profile of the Chimaera^TM^ long cephalomedullary nail for the treatment of extracapsular proximal femoral fractures in adult patients. Eventual bone union was clinically documented in all evaluable patients (N = 42, 100%), with early consolidation already recognized at the first follow-up (~1.3 months) in 16 cases, and the median time to documented union was 7.4 months. Although follow-up occurred beyond the planned 9-15-month window in several cases, this was interpreted as delayed availability of documentation of bone union rather than evidence of biologically delayed fracture healing.

The exclusion of two patients from the primary endpoint assessment should be considered when interpreting the union rate; however, given the absence of documented treatment failure in these patients, it is unlikely that this aspect substantially affected the assessment of primary objective achievement. Nevertheless, including 42 evaluable patients rather than the 44 originally planned participants resulted in greater residual statistical uncertainty, as reflected by the lower bound of the 95% confidence interval. Safety assessed in the FAS (44/44) was also favorable, with four adverse events (9.1%) and two re-operations (4.5%) reported, which were unrelated to the investigation device. The femoral shaft fracture occurring in the distal locking region and the deep infection, in particular, were evaluated as unrelated to device performance.

The eventual union rate observed in our cohort was consistent with rates reported in the literature and with the 98% baseline reference considered during the study design phase [11-13,18], but the small sample size and the range of the 95% confidence interval preclude solid inference of superiority. This result is consistent with current evidence supporting the use of CMNs for extracapsular proximal femoral fractures, yet specifying that implant choice remains debated depending on fracture patterns [11,19]. Interestingly, union was clinically documented in all patients despite an elderly cohort in which ~38% had osteoporosis - features typically associated with higher fixation-failure risk [20]. Although the observed time to bone consolidation exceeded that reported in other studies [21,22], we point out that 38.1% of patients in our cohort already achieved union at the first follow-up. This result should be evaluated considering the retrospective design of our study, where the time to union mirrors the first documented confirmation. This implies that our median time to consolidation may have overestimated the actual biological consolidation time because of variable imaging availability and out-of-window visits. However, the lack of a concurrent control group limits comparative inference.

The mean operative time in our study (91.3 ± 45.7 minutes) was interestingly lower than the ~119 minutes reported in a prior prospective study on Chimaera^TM^ long nail [23], yet still above the times described for other nails (~60 minutes) [24]. This pattern possibly suggests both a learning-curve effect concerning application of the system and design-specific steps that may lengthen early procedures, possibly indicating potential for further optimization as familiarity with the system increases [23].

Device-related complications remain the principal concern in CMN application and still represent a target for ongoing technical optimization [14,15]. Implant-related fractures or screw cutouts, usually listed among the most frequent device-associated events [11,24], were not observed in our analysis. Even though we cannot exclude possible late complications, which were not evaluated, and the missed detection of minor asymptomatic events, safety results may reflect both growing surgical experience and beneficial design attributes of the system. Advantageous designing features could be further supported by TAD values observed in the study, which were over 25 mm at the first follow-up and apparently decreased over time, reaching 24.7 ± 4.8 mm at the third follow-up visit. A 25 mm cut-off (originally proposed for sliding hip screws) is frequently referred to as a predictor of lag screw cut-out [25]. Although the 25 mm limit has also been confirmed in recent reports [26], the specificity of this threshold limit exceeds sensitivity, and some authors have proposed different cut-off values [27]. In our cohort, no screw cut-out was observed despite TAD > 25 mm in some cases, possibly reflecting the combined impact of reduction quality, screw positioning within the femoral head, bone quality, and implant design [28]. While this result should indeed be interpreted cautiously because postoperative and late follow-up measurements were based on different sample sizes and no paired analysis was performed, several features of the construct and its usage may underpin the observed outcomes. The telescoping lag screw supports controlled fracture-site compression under weight bearing, and the frequent use of the supplementary anti-rotational screw may enhance rotational stability of the head-neck fragment in osteoporotic bone, fostering consolidation. CMNs with a second anti-rotational element have been associated with reduced complications and improved clinical/functional outcomes [29] and are particularly advocated for AO 31-A patterns [30], which mirrored the fracture distribution in this study. Systematic distal locking - predominantly in static mode - likely prioritized primary stability while preserving the option for biologic impaction if healing lagged. The exclusive adoption of long nails (280-420 mm), spanning the diaphysis, may have mitigated tip-related peri-implant fractures, none of which were recorded as device-related events in our cohort.

Overall, results should be interpreted considering methodological constraints inherently related to the study design. Given the retrospective nature of the investigation, follow-up schedules varied substantially, with a portion of assessments conducted via telephone and many occurring outside the predefined time windows. This may have led to underestimation of minor complications and delayed recognition of radiographic consolidation, as imaging was not always available. The absence of a concurrent control group limits comparative inference versus alternative CMN designs or lengths. Moreover, data could be biased by the exclusive use of long nails (280-420 mm), the high uptake of the supplementary anti-rotational screw (86.4%), treatment by surgeons experienced with the system, and a cohort considering Caucasian subjects exclusively.

## Conclusions

This retrospective post-market study provides descriptive real-world data on the use of the Chimaera^TM^ long nail for extracapsular proximal femoral fractures. Eventual bone union was clinically documented in all evaluable patients, and a few adverse events were recorded, none related to the nailing system, with no implant-related mechanical complications.

While these findings possibly suggest that the design characteristics of the system may contribute to stable fixation and reliable fracture healing in a real-world scenario, given the retrospective design, small sample size, incomplete and heterogeneous radiographic follow-up, and absence of a comparator group, data should be interpreted cautiously and require confirmation in prospective controlled studies.
